# Secondary Primary Malignancies in Multiple Myeloma: An Old Nemesis Revisited

**DOI:** 10.1155/2012/801495

**Published:** 2012-07-19

**Authors:** Jay Yang, Howard R. Terebelo, Jeffrey A. Zonder

**Affiliations:** ^1^Department of Oncology, Karmanos Cancer Institute and Wayne State University, Detroit, MI 48201, USA; ^2^Department of Internal Medicine, Providence Hospital, Southfield, MI 48075, USA

## Abstract

The treatment of myeloma has undergone extraordinary improvements in the past half century. These advances have been accompanied by a concern for secondary primary malignancies (SPMs). It has been known for decades that extended therapy with alkylating chemotherapy agents, such as melphalan, carries an increased risk of therapy-related myelodysplastic syndrome and/or acute myeloid leukemia (t-MDS/AML), with a cumulative risk as high as 10–15%. High-dose chemotherapy with autologous stem cell support became widely accepted for myeloma in the 1990s. Despite the use of high doses of melphalan, the risk of t-MDS/AML with this procedure is estimated to be less than 5%, with much of this risk attributable to pretransplant therapy. Recently, lenalidomide has come under scrutiny for its possible association with SPMs. It is too soon to declare a causal relationship at this time, but there appears to be an increased number of SPMs in reports from several studies using lenalidomide maintenance. Current studies should be amended and future studies planned to better define the risk of SPMs and the risk factors and mechanisms for its development. Patients should be educated regarding this potential concern but the current use of lenalidomide should not generally be altered until further data are available.

## 1. Introduction

The evolution of myeloma therapy has been one of the success stories in the fight against cancer. Current treatment options for myeloma include melphalan, other cytotoxic agents, corticosteroids, high-dose therapy with autologous stem cell transplant (HDT/SCT), and more recently, novel agents such as bortezomib, thalidomide, and lenalidomide. Despite the remarkable improvement in prognosis for myeloma patients, the disease remains incurable and is characterized by multiple relapses. Therapy-related myelodysplasia and acute myeloid leukemia (t-MDS/AML) have been recognized as a consequence of treatment with alkylating agents and/or anthracyclines. Similar concerns have now been raised about the potential for an increase in secondary primary malignancies (SPMs) in myeloma patients exposed to lenalidomide, and, in particular, long-term exposures in a maintenance setting.

In this paper, we will review the developmental history of myeloma therapy, with particular emphasis on the risk of secondary cancers, and examine the available data with regard to the risk of SPMs seen with lenalidomide. We also speculate about the mechanism(s) by which lenalidomide could increase the risk of second cancers. To conclude, we make some recommendations about how our current understanding affects our treatment decisions and suggest directions for future research. As new data emerge about lenalidomide and the risk of SPMs, it is our hope that this paper will help to put that information in proper perspective.

## 2. Second Cancers in Multiple Myeloma

 SPMs are not an uncommon occurrence among cancer patients. The NCI's SEER program analyzed its database from 1973 to 2000 and reported that the cumulative incidence of SPMs was nearly 14% at 25 years of followup for cancer patients in general [1]. Myeloma patients had a 6.1% incidence of SPMs at 20 years but the overall rate was not higher than that seen in the general population. However, increased relative risks for AML, chronic myelogenous leukemia, and Kaposi's sarcoma were noted. Leukemias, especially AML, accounted for the largest cancer excesses and likely reflected treatment with alkylating agents.

The Finnish Leukaemia Group conducted a retrospective, long-term followup of 432 patients who were treated with conventional chemotherapy for myeloma [2]. The number and distribution of secondary solid cancers were similar to the general population but the actuarial risk of leukemia was almost 10% at 9 years. A Swedish registry database that included 8656 myeloma patients found a 5.5% risk of SPMs [3]. According to their analysis, myeloma patients had a marked increased risk of AML (standardized incidence ratio, that is, ratio of observed/expected rates = 8.19; 95% CI, 5.7–11.4), a slight increased risk of non-Hodgkin lymphoma (NHL) (SIR = 1.74; 95% CI, 1.12–2.57), and a decreased risk of solid cancers (SIR = 0.81; 95% CI, 0.73–0.90). In summary, based on older registry data, an increased risk of AML is observed in myeloma patients.

## 3. Chemotherapy for Multiple Myeloma

 The use of chemotherapy for myeloma began in 1962 when melphalan was first reported to have activity in this disease [4]. Many patients were treated with continued courses of melphalan indefinitely until disease progression or unacceptable toxicity [5]. Combinations that added other chemotherapeutic agents such as vincristine, carmustine, doxorubicin, or nitrosureas to a melphalan/prednisone backbone showed somewhat higher response rates but no survival benefit [6–8]. Regardless of therapy given, all patients relapsed and so the concept of maintenance therapy became attractive with the goal of prolonging remissions. Trials that tested maintenance chemotherapy after responses to melphalan-based induction showed no clinical or survival benefit over observation alone [9]. Patients receiving maintenance had slightly longer remission durations which were offset by lower rates of second remissions, suggesting that maintenance therapy contributed to drug resistance at the time of relapse [10].

The practice of indefinite melphalan therapy also came under scrutiny because of a burgeoning worry about secondary malignancies. Kyle et al. were among the first to propose an association between the prolonged use of melphalan and myelodysplasia and/or acute myeloid leukemia (MDS/AML) [11]. Some questioned whether MDS/AML could be part of the natural history of myeloma, much like what is seen in other hematological conditions. Although this view was buoyed by several reported cases of untreated myeloma and concurrent AML in the literature [12, 13], the prevailing conclusion was that such cases represented a chance association. With time, MDS and AML became recognized complications after chemotherapy for other neoplasms such as Hodgkin lymphoma (HL) and ovarian cancer, and it became accepted that, regardless of the indication, chemotherapy was directly responsible for this increased risk due to mechanisms that included direct DNA damage.

Reported rates of t-MDS/AML for myeloma patients treated with melphalan ranged from anywhere from 3% at 5 years and 10% at 8 years, with estimates as high as 25% at 10 years [14]. Higher cumulative doses of melphalan increased the risk of MDS/AML [15]. Cyclophosphamide also appeared to carry a risk, but less so than melphalan [16]. The diagnosis of t-MDS/AML carried a grim prognosis with reported median survivals of less than 3 months [2, 11]. Nevertheless, the use of melphalan remained the *de facto* standard for several decades.

## 4. HDT/SCT for Multiple Myeloma

The use of autologous bone marrow transplantation with high-dose melphalan to treat myeloma became widely accepted as the standard of care for transplantable patients after the IFM 90 trial showed event-free and overall survivals in favor of this procedure [17]. The use of HDT/SCT for both solid tumors and hematologic malignancies, including myeloma, increased dramatically in the 1990s [18]. Cases of MDS/AML were seen after HDT and raised serious concerns about the leukemogenic risks of the procedure.

The risk of t-MDS/AML after HDT/SCT was first more clearly defined in the lymphoma patient population. Patients transplanted at the University of Minnesota for lymphoma experienced a 14.5% cumulative incidence of t-MDS/AML [19]. The risk increased with patient age and with the burden of alkylating agents prior to transplant [20]. Forrest et al. reported a 15-year cumulative incidence of 11% that indicated not only an increased risk of MDS/AML (relative risk = 47.2), but also lymphoproliferative disoders (RR = 8.1) and solid tumors (RR = 1.98) [21]. Interestingly, this analysis included 800 patients who were autotransplanted for a variety of conditions but there were no reported cases of MDS/AML in the subset of 123 myeloma patients.

 Given the historical experience with melphalan-associated t-MDS/AML, concern arose over the risk in myeloma patients treated with high-dose melphalan. Govindarajan et al. reviewed 188 pts with myeloma who underwent HDT/SCT at the University of Arkansas [22]. In 117 patients who received extended courses of chemotherapy prior to tandem autotransplantation, 7 cases of MDS were seen, whereas in 71 patients who received limited chemotherapy prior to transplantation, no cases of MDS were seen. They concluded that preceding therapy was likely the cause of MDS in most cases seen after HDT/SCT, a finding that mirrored conclusions from studies in Hodgkin lymphoma (HL) [23].

The Arkansas experience was reviewed again over a decade later, this time including 3077 patients undergoing HDT/SCT for myeloma, most of whom were treated on their total therapy or total therapy-like protocols [24]. MDS-associated cytogenetic abnormalities were seen in 6%, although in roughly 2/3 of these cases, the karyotypic changes were only transient. The risk of clinically overt MDS/AML was even less, estimated at only 1% of transplanted patients. Survival after the diagnosis of t-MDS/AML in transplanted patients has been poor in most studies with a median of about 6 months [20].

In summary, contemporary studies show the rate of t-MDS/AML after HDT/SCT for myeloma to be less than 5% with higher rates in cohorts that received more previous alkyating-containing therapy. This risk is lower than in most reported series for lymphoma, which can be explained, in part, by the earlier use of the transplant in myeloma, the emphasis on avoiding pretransplant stem cell damaging agents, and the abrogated use of total body irradiation during conditioning [25].

## 5. Lenalidomide Development and Mechanisms of Action

 The immunomodulatory drugs (IMIDs) represent a novel class of antineoplastic agents that include thalidomide and its congeners, lenalidomide (CC-5013) and pomalidomide (CC-4043) [26]. Lenalidomide has significant activity versus myeloma and is approved as treatment in combination with dexamethasone. Lenalidomide has also been shown to have activity in a wide variety of other hematological malignancies, including MDS, non-Hodgkin lymphoma, HL, chronic lymphocytic leukemia, and myelofibrosis. It has an indication for transfusion-dependent anemia due to low or intermediate-1 risk MDS associated with a deletion 5q cytogenetic abnormality. Although approved in the United States for MDS, the European Medicines Agency (EMEA) has not yet granted approval for this indication due to initial safety concerns—namely the risk of progression to AML [27]. Recent evidence, however, including a randomized trial, has not shown an increased risk of AML in MDS patients treated with lenalidomide [28, 29].

 The story of lenalidomide's development, broad antitumor activity, and drug approval is remarkable considering its unclear mechanism of action. Lenalidomide has a wide variety of effects, including antiangiogenesis and modulation of the tumor microenvironment, but it appears that its direct tumoricidal and immunomodulatory properties have the most relevance in myeloma [30, 31]. Its tumoricidal properties appear to be mediated in part by inhibiting the myeloma survival factor, IRF4, resulting in cell cycle arrest [32], as well as caspase-mediated apoptosis [33]. Its immunomodulatory properties include stimulation of immune effector cells such as NK and T cells [34]. The precise molecular target of lenalidomide in multiple myeloma has been elusive [35], but recently cereblon has been identified [36, 37]. The potential molecular targets in MDS have been reviewed elsewhere [38].

 Thus, while lenalidomide itself is a simple compound, its molecular effects are pleiotropic and rather poorly understood. Furthermore, the relative contribution of these different mechanisms to its antimyeloma activity has not been well characterized [39] and may depend on multiple factors including the tumor type, dose/duration/schedule of lenalidomide, concomitant drugs given, and other preexisting patient factors.

## 6. Lenalidomide Therapy and Concern for SPMs

Due to disappointing results observed with maintenance chemotherapy, alternatives were eagerly sought. Interferon [40, 41] and corticosteroids [42, 43] were each tested in multiple clinical trials but were never convincingly shown to be beneficial [44]. Novel agents, with their unprecedented activity, became attractive candidates for use in the maintenance setting.

Thalidomide is the best studied of the novel agents for post-SCT maintenance, where it has prolonged progression-free survival (PFS) and/or time to progression (TTP) in multiple studies [45–49]. Despite this, only 2 studies have demonstrated a survival benefit, which may be partially explained by the shorter survivals seen after relapse in some studies [45, 49, 50]. This has again raised concerns about the selection of resistant clones at the time of relapse or progression [51]. The use of thalidomide as maintenance has never become routine by most prescribing clinicians due to its side effect profile and lack of consistent mortality benefit.

Both lenalidomide and bortezomib are being evaluated as maintenance therapy. For upfront and relapsed disease, both agents offer high levels of activity with unique but favorable side effect profiles. Lenalidomide is given orally, generally lacks the cumulative neuropathic potential of either thalidomide or bortezomib, and thus, may be the most promising drug in this setting. Combinations of novel agents are also being studied as maintenance [52, 53].

In the era of novel agents, second malignancies had been overlooked as a serious concern. Three recently published studies, however, provoked substantial interest due to the reported increased risk of SPMs (Table 1). All three were phase 3, placebo-controlled, randomized trials testing lenalidomide as maintenance therapy, either after HDT/SCT (IFM 2005-002, CALGB 100104) or after induction therapy (MM-015). These studies are of critical importance since they represent the first and only reported randomized trials to date that have prospectively and intentionally measured SPMs in multiple myeloma patients treated with maintenance lenalidomide versus placebo.

In the IFM 2005-002 study, patients under the age of 65 years with nonprogressive disease after HDT/SCT received 2 cycles of consolidation lenalidomide and then were randomized to either maintenance lenalidomide (10–15 mg daily) or placebo [54]. Lenalidomide improved median progression-free survival from 23 months to 41 months (HR = 0.5, *P* < 0.001). The 4-year overall survival was about 75% in both arms. The incidence of SPMs was 3.1 per 100 patient-years and 1.2 per 100 patient-years for patients receiving lenalidomide and placebo, respectively (*P* = 0.002). There were 13 reported hematological cancers with lenalidomide and 5 with placebo but the numbers of MDS/AML were similar (5 versus 4). Surprisingly, 7 cases of acute lymphoblastic leukemia or Hodgkin lymphoma were recorded in the lenalidomide maintenance arm and none in the placebo arm. Since all patients had received at least 2 years of lenalidomide and the optimal duration of maintenance is not known, the IFM has elected to stop the trial for safety reasons, discontinuing lenalidomide in the remaining patients still in remission.

 In the CALGB 100104 study, patients under the age of 70 years with stage I-III myeloma were given induction therapy followed by HDT/SCT [55]. Those with stable disease or better were then randomized at day 100–110 posttransplant to lenalidomide (10–15 mg daily) or placebo until progression. The estimated median TTP was 46 months for lenalidomide and 27 months for placebo, results that are similar to the IFM study. The cumulative incidence of SPMs was 8% in the lenalidomide maintenance arm versus 3% in the placebo arm, but even more striking, there were 8 cases of hematological cancers (including 6 with MDS/AML) seen with lenalidomide and only 1 with placebo. This study has now also reported a survival benefit with maintenance lenalidomide.

 MM-015 randomized older patients to 1 of 3 arms: MPR-R (melphalan, prednisone, and lenalidomide induction followed by maintenance lenalidomide), MPR (melphalan, prednisone, and lenalidomide induction), or MP (melphalan and prednisone induction) [56]. After 9 cycles of induction therapy, the MPR-R arm received maintenance lenalidomide at 10 mg for 21 of every 28 days, while the other two arms received placebo maintenance. Median PFS was significantly longer with MPR-R (31 months) compared with MPR (14 months) and MP (13 months). No significant survival differences were seen. There were 12 cases of SPM in the MPR-R arm, 9 cases in the MPR arm, and 4 cases in the MP arm [57]. Ten cases of MDS/AML were seen in the lenalidomide containing arms (incidence 2.6%) and 1 case in the MP arm (0.6%). The number of solid tumors was low with no major differences seen between groups.

 Several other reports have offered long-term, albeit post hoc, safety data for lenalidomide (Table 2) [58–60]. All have reported relatively low numbers of SPMs, including MDS/AML, with incidence rates ranging from 1.5 to 7.4%. The observed rates of SPMs were generally no higher than expected based on historical SEER data. An analysis of pooled data from 11 industry-sponsored trials suggested that there was no correlation between the development of SPMs and the cumulative dose or duration of lenalidomide received [58]. An analysis of the randomized MM 009/010 study noted higher rates of nonmelanoma skin cancers in patients randomized to lenalidomide compared with placebo [58]. There were no SPMs reported after the discontinuation of protocol treatment leading the authors to conclude that there are considerable obstacles in the ascertainment of second cancers during long-term followup.

## 7. Discussion

At the moment, there are more questions than answers, so we have designed our discussion around some of the most relevant issues.

### 7.1. Is There a True Risk of SPMs with Lenalidomide?

When overall risks are relatively small, as they appear to be with SPMs in myeloma, it becomes more difficult to make conclusions with certainty. A number of other practical and statistical limitations exist when analyzing the data. SPMs may be underestimated if they are not specifically tracked during followup, particularly if off study. On the other hand, overreporting or overdiagnosis of SPMs may occur if they are expected on treatment arms. In retrospective or post hoc analyses, the methods of data collection may be less than desired. Finally, several reports have compared observed rates of SPMs to nonrandomized cohorts, which can sometimes be misleading. Some of these concerns are minimized when analyzing results from randomized, placebo-controlled trials with an *a priori* intention to measure SPMs. However, crossover, either on or off study, still has the potential to confound results. In the CALGB 100104 trial, the majority (67%) of the patients in the placebo arm crossed over to lenalidomide after unblinding. In the IFM 2005-002 and MM-015 trials, patients remained on their assigned treatment after unblinding, but it is likely that many of them received salvage lenalidomide at the time of progression.

Despite these limitations, there are some potentially important observations that merit attention and will need validation. The cumulative rates of invasive SPMs in the lenalidomide arms across the 3 randomized studies were quite consistent, ranging from 7 to 7.8%, thereby strengthening the observation. All 3 studies reported increased numbers of hematological malignancies in the lenalidomide arms and, in particular, 2 of the studies reported increased numbers of MDS/AML. The reported solid cancers have been heterogeneous in type. The other reviewed studies report long-term outcomes for cohorts of patients treated with lenalidomide. These studies have indicated a low risk of SPMs with rates that are no more than to be expected based on SEER data.

In aggregate, the retrospective and registry data does not support an increased risk of SPMs with chronic lenalidomide therapy. However, the recently published randomized trials demonstrate a signal suggesting otherwise. The risk of developing hematological malignancies, including MDS/AML, appears to be greater than the risk of solid cancers.

### 7.2. What Are the Risk Factors for the Development of SPMs?

Treatment-related risk factors have received the most attention to date. There is certainly the strong possibility of an interaction between exposure to melphalan, exposure to lenalidomide, and an increased risk of MDS/AML. In the aforementioned randomized studies, patients were given melphalan either as induction therapy or as part of HDT/SCT prior to maintenance lenalidomide. There are no data at this time to suggest that an increased duration or dose of lenalidomide corresponds to an increased risk of SPMs. Whether the leukemogenicity of other chemotherapeutic agents is potentiated by lenalidomide remains to be seen. For example, results of the IFM 2005-002 trial have suggested an increased number of hematological cancers in those who received either a tandem transplant or pretransplant chemotherapy that included cyclophosphamide, etoposide, and cisplatin.

Non-treatment-related factors are less well understood but may play significant roles. Potential disease-related risk factors include baseline complex cytogenetics and the subtype of myeloma. The MM-015 study noted that 3 of the patients who ultimately developed MDS/AML in the lenalidomide arm were part of a small group of 11 patients who had complex cytogenetics at baseline [57]. IgG and IgA isotype MGUS patients have been reported to have an increased risk of MDS/AML [61]. Host factors, such as genetic polymorphisms [62], environmental factors, and behavioral factors have also been postulated as risk factors.

### 7.3. What Are the Potential Causal Mechanisms of Lenalidomide-Associated SPMs?

A truly satisfactory explanation would require a better understanding of how lenalidomide works in patients with hematologic malignancies, let alone myeloma. This is an area of active research, but certainly more needs to be done. Unlike traditional cytotoxic chemotherapies, lenalidomide showed no mutagenic potential in extensive genotoxicity studies performed during its development [27]. And although carcinogenicity testing was not performed, chronic studies in rat and monkey revealed no potential for tumorigenicity. However, lenalidomide is clearly myelosuppressive and tumoricidal, and after a prolonged exposure may affect the ability to mobilize and collect stem cells. Perhaps these properties are an indication of a myelotoxicity which may predispose to MDS or AML. One could also speculate that lenalidomide's immunomodulatory properties and its effects on the tumor microenvironment may allow for the propagation of abnormal clones which can result in a malignancy. The complex mechanisms of action responsible for lenalidomide's activity, as well its unclear molecular target, make it difficult to rely on preclinical data to assess the safety and neoplastic potential of lenalidomide.

### 7.4. What Does the Myeloma Research Community Need to Do Now?

 There is a great need for additional systematic data gathering to determine whether lenalidomide is truly associated with SPMs, and if so, what types. For studies that are ongoing, amendments should be made to the protocols to include enhanced monitoring and precise measurements of second cancers. Careful monitoring for skin cancers may be important as the MM009/010 study has reported increased numbers on the lenalidomide arm. Although the large majority of skin cancers are found at an early and treatable stage, a finding of increased skin cancers would represent a proof-of-principle of the cancer promoting potential of lenalidomide.

Prospective randomized studies of lenalidomide versus placebo that include SPMs as a well-defined endpoint would be ideal. However, studies of this nature may be difficult to plan now that lenalidomide has already been established as an effective standard therapy for myeloma. However, the IMIDs, including pomalidomide, are also being evaluated in a variety of other malignancies as well as nonmalignant autoimmune mediated disorders. Careful monitoring for SPMs should be incorporated into these trials. Future protocols should include bone marrow examinations with cytogenetic analyses as part of routine monitoring.

Careful statistical analyses of the accumulating data will be critical. With the risk of SPMs being relatively low, small numbers of reported SPMs could significantly alter the results. We recommend that incidence rates of SPMs be adjusted for person-years at risk (i.e., rate per 100 person-years). This mitigates the possibility of overestimating the risk of SPMs in lenalidomide-treated patients due to longer patient survival.

 Clinical and preclinical research is needed to better elucidate lenalidomide's mechanism of action and its potential role in secondary cancers. The following is a partial list of important questions to be resolved.

- Is there a relationship between the amount of lenalidomide exposure (dose, duration, or schedule) and the risk of SPMs?
- Do baseline cytogenetic abnormalities increase the risk of developing SPMs?
- Is patient age or previous history of malignancy a risk factor for the development of SPMs?
- Are there other treatment (e.g., type of chemotherapy), host (e.g., SNPs), or disease (e.g., genetic aberrations) related risk factors or biomarkers for the development of SPMs?
- What are the characteristics, prognosis, and natural history of these SPMs? What is the time to development? For t-MDS/AML, what types of cytogenetic and molecular changes are seen and are they different than the pattern already established in cases due to cytotoxic chemotherapy or radiation?

### 7.5. Does This Change How We Treat Our Myeloma Patients?

 In our opinion, there is not enough evidence at this time to conclude that lenalidomide definitively increases the risks of second cancers, but there is a cause for concern. Patients should be informed of the potential increased risk of SPMs and an informed decision should be made keeping in mind the risks and benefits. The benefits of lenalidomide therapy for active disease in the upfront and relapsed settings are well-documented and include better and deeper responses, longer progression-free survival, and longer overall survival compared with older standard therapies [63, 64]. The information we have now should not drastically alter the decision-making process for the majority of these patients.

**Figure 1:**
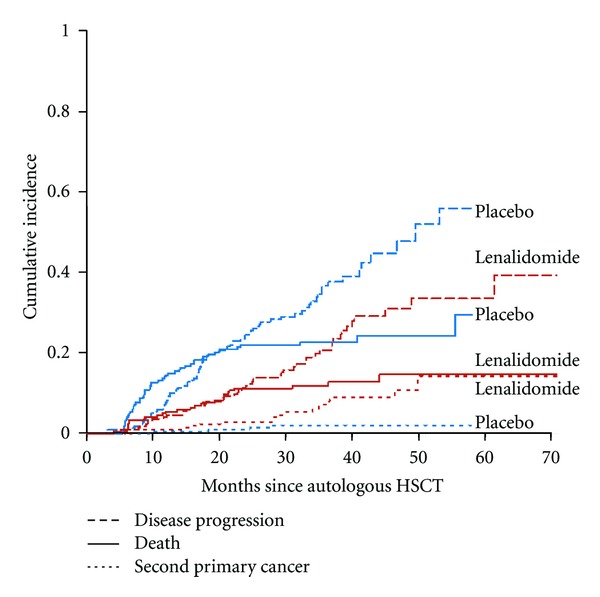
Competing risks in multiple myeloma patients. Cumulative incidence of second primary cancers, disease progression, and death for patients randomized to either maintenance lenalidomide or placebo post HDT/SCT in the CALGB 100104 trial. In placebo treated patients (in blue), the risk of death and disease progression far exceeds the risk of SPM. Reprinted with permission [55].

In the maintenance setting, the risk-benefit analysis may be more complex. The risks of extended lenalidomide therapy not only include SPMs but also well-known toxicities such as myelosuppression, fatigue, and thrombosis. Studies have consistently demonstrated that lenalidomide maintenance results in sizable improvements in disease control. The CALGB 100104 study has also recently shown a survival benefit—an exciting result that has not been confirmed yet in other studies. The risks of SPMs related to lenalidomide, if any, are currently poorly defined but may be estimated to be about 7%, for the purposes of discussion. As such, it should be kept in mind that for most myeloma patients, the competing risks of death due to disease progression exceed the risk of SPMs (Figure 1) [65]. A post hoc analysis of the CALGB 100104 trial included SPMs as primary events (along with disease progression and death) and still showed an impressive improvement in EFS (HR 0.53, 95% CI, 0.41–0.69) for patients on maintenance lenalidomide compared with placebo. 

One situation which may tilt the physician/patient discussion against lenalidomide maintenance is when the patient has a known genetic predisposition to cancer, such as BRCA, or a strong personal or family history of cancer. Although there is no firm data in this regard and we do not feel that this completely precludes the use of lenalidomide as maintenance, it has been our experience that such patients are reluctant to pursue this strategy and prefer to reserve the use of this drug until the time of progression.

We do recommend that all patients starting lenalidomide maintenance have a baseline bone marrow examination with cytogenetics to ensure that there is no overt evidence of dysplasia or concerning cytogenetic abnormalities. There should be a low threshold for a careful bone marrow analysis with karyotyping for patients with unexplained cytopenias that persist despite lenalidomide withdrawal. Patients should undergo age-appropriate cancer screening measures and clinicians should have a high index of suspicion when evaluating patient symptoms or findings that may represent a second malignancy.

## 8. Final Thoughts

 Lenalidomide is an exciting drug with an impressive range and depth of activities. With the ever-expanding investigation and application of lenalidomide in myeloma and other hematological malignancies, there is a substantial need to define its possible contribution to SPMs. This concern is warranted but has come about, in large part, due to the significant improvements in survival seen in myeloma patients. The myeloma research community has much work to do to shed light on this important issue. In the meantime, our general frame-of-mind is quite similar to that of Kyle et al. who presciently opined some 35 years ago, “Late death after a long remission of myeloma is much to be preferred to early death without remission” [66].

## Figures and Tables

**Table 1:** Incidence of SPMs with lenalidomide (Len) maintenance versus placebo in prospective, randomized phase 3 trials.

Trial	Reference	*N*	F/u^a^ (mo)	Treatment prior to randomization	Randomized arms	Invasive SPMs (no.)	Solid cancers (no.)	Heme cancers (no.)	Nonmelanoma skin cancers (no.)	Cumulative incidence of invasive cancers (%)	Incidence (per 100 patient-years)
IFM 2005-002	[54]	614	45	Induction then SCT	Len	23	10	13	5	7.5	3.1
					Placebo	9	4	5	3	2.9	1.2

CALGB 100104	[55]	460	34	Induction then SCT	Len	18	10	8	4	7.8	N/A
					Placebo	6	5	1	3	2.6	N/A

					MPR-R	12	5	7	1	7	1.4
MM-015	[56, 57]	459	30	MPR or MP	MPR	9	4	5	4	7	2.1
					MP	4	3	1	5	3	0.7

^a^ Median followup from the time from randomization.

**Table 2:** Incidence of SPMs with lenalidomide (Len)-based therapy in selected trial cohorts based on retrospective or post hoc analyses.

Trial/Location	Reference	*N*	F/u	Patient population	Treatment	Invasive SPMs	Cumulative incidence of SPMs^a^ (%)	IR^b^	SIR^c^ (95% CI)	Types (number of cases)
PMH	[60]	230	N/A	RRMM^d^	Len-based	6^f^	2.6	Not reported	Not reported	MDS/AML
Cornell	[59]	68	>5 yrs	NDMM^e^	BiRD^g^	5	7.4^h^	2.85	1.36	MDS/AML (0), solid (5)
MM009-010	[58]	704	48 mo	RRMM	Len/dex	8	2.3	1.71	N/A	MDS (2), AML (0), solid (6), B-cell (0)
Pooled data	[58]	3839	N/A	RRMM	Len-based	57	1.5	2.35	0.77 (0.43–1.28)	MDS (8), AML (1), B-cell (2), solid (46)

^a^Excluding nonmelanoma skin cancers.

^b^Incidence rate per 100 person-years at risk.

^c^Standardized incidence ratio (i.e., ratio of observed/expected rates).

^d^Relapsed and/or refractory multiple myeloma.

^e^Newly diagnosed multiple myeloma.

^f^Only MDS/AML was reported.

^g^Clarithromycin, lenalidomide, and dexamethasone.

## References

[1] Curtis RE, Freedman DM, Ron E (2006 ). New malignancies among cancer survivors: SEER cancer registries, 1973–2000. *NIH Publication*.

[2] Olivanen T (2000). Acute leukaemia and other secondary neoplasms in patients treated with conventional chemotherapy for multiple myeloma: a Finnish Leukaemia Group study. *European Journal of Haematology*.

[3] Dong C, Hemminki K (2001). Second primary neoplasms among 53 159 haematolymphoproliferative malignancy patients in Sweden, 1958–1996: a search for common mechanisms. *British Journal of Cancer*.

[4] Bergsagel DE, Sprague CC, Austin C, Griffith KM (1962). Evaluation of new chemotherapeutic agents in the treatment of multiple myeloma—IV. L-Phenylalanine mustard (NSC-8806). *Cancer Chemotherapy Reports. Part 1*.

[5] Hoogstraten B, Sheehe PR, Cuttner J (1967). Melphalan in multiple myeloma. *Blood*.

[6] MacLennan ICM, Cusick J (1985). Objective evaluation of the role of vincristine in induction and maintenance therapy for myelomatosis. *British Journal of Cancer*.

[7] Oken MM, Harrington DP, Abramson N, Kyle RA, Knospe W, Glick JH (1997). Comparison of melphalan and prednisone with vincristine, carmustine, melphalan, cyclophosphamide, and prednisone in the treatment of multiple myeloma: results of Eastern Cooperative Oncology Group Study E2479. *Cancer*.

[8] Blade J, San Miguel JF, Alcala A (1993). Alternating combination VCMP/VBAP chemotherapy versus melphalan/prednisone in the treatment of multiple myeloma: a randomized multicentric study of 487 patients. *Journal of Clinical Oncology*.

[9] Alexanian R, Balcerzak S, Haut A (1975). Remission maintenance therapy for multiple myeloma. *Archives of Internal Medicine*.

[10] Belch A, Shelley W, Bergsagel D (1988). A randomized trial of maintenance versus no maintenance melphalan and prednisone in responding multiple myeloma patients. *British Journal of Cancer*.

[11] Kyle RA, Pierre RV, Bayrd ED (1970). Multiple myeloma and acute myelomonocytic leukemia. *New England Journal of Medicine*.

[12] Rosner F, Gruenwald H (1974). Multiple myeloma terminating in acute leukemia. Report of 12 cases and review of the literature. *American Journal of Medicine*.

[13] Cleary B, Binder RA, Kales AN, Veltri BJ (1978). Simultaneous presentation of acute myelomonocytic leukemia and multiple myeloma. *Cancer*.

[14] Dispenzieri A, Lacy MQ, Greipp PR, Greer JP, Foerster J, Rodgers GM (2009). Multiple myeloma. *Wintrobe's Clinical Hematology*.

[15] Cuzick J, Erskine S, Edelman D, Galton DAG (1987). A comparison of the incidence of the myelodysplastic syndrome and acute myeloid leukaemia following melphalan and cyclophosphamide treatment for myelomatosis. *British Journal of Cancer*.

[16] Greene MH, Harris EL, Gershenson DM (1986). Melphalan may be a more potent leukemogen than cyclophosphamide. *Annals of Internal Medicine*.

[17] Attal M, Harousseau JL, Stoppa AM (1996). A prospective, randomized trial of autologous bone marrow transplantation and chemotherapy in multiple myeloma. *New England Journal of Medicine*.

[18] Pasquini MC, Wang Z Current use and outcome of hematopoietic stem cell transplantation: CIBMTR Summary Slides. http://www.cibmtr.org.

[19] Miller JS, Arthur DC, Litz CE, Neglia JP, Miller WJ, Weisdorf DJ (1994). Myelodysplastic syndrome after autologous bone marrow transplantation: an additional late complication of curative cancer therapy. *Blood*.

[20] Pedersen-Bjergaard J, Andersen MK, Christiansen DH (2000). Therapy-related acute myeloid leukemia and myelodysplasia after high-dose chemotherapy and autologous stem cell transplantation. *Blood*.

[21] Forrest DL, Nevill TJ, Naiman SC (2003). Second malignancy following high-dose therapy and autologous stem cell transplantation: incidence and risk factor analysis. *Bone Marrow Transplantation*.

[22] Govindarajan R, Jagannath S, Flick JT (1996). Preceding standard therapy is the likely cause of MDS after autotransplants for multiple myeloma. *British Journal of Haematology*.

[23] Harrison CN, Gregory W, Vaughan Hudson G (1999). High-dose BEAM chemotherapy with autologous haemopoietic stem cell transplantation for Hodgkin’s disease is unlikely to be associated with a major increased risk of secondary MDS/AML. *British Journal of Cancer*.

[24] Barlogie B, Tricot G, Haessler J (2008). Cytogenetically defined myelodysplasia after melphalan-based autotransplantation for multiple myeloma linked to poor hematopoietic stem-cell mobilization: the Arkansas experience in more than 3000 patients treated since 1989. *Blood*.

[25] Moreau P, Facon T, Attal M (2002). Comparison of 200 mg/m2 melphalan and 8 Gy total body irradiation plus 140 mg/m2 melphalan as conditioning regimens for peripheral blood stem cell transplantation in patients with newly diagnosed multiple myeloma: final analysis of the Intergroupe Francophone du Myélome 9502 randomized trial. *Blood*.

[26] Bartlett JB, Dredge K, Dalgleish AG (2004). The evolution of thalidomide and its IMiD derivatives as anticancer agents. *Nature Reviews Cancer*.

[27] European Medicines Agency Withdrawal assessment report for lenalidomide Celgene Europe. http://www.ema.europa.eu/docs/en_GB/document_library/Application_withdrawal_assessment_report/2010/01/WC500065821.pdf.

[28] Adès L, Le Bras F, Sebert M (2012). Treatment with lenalidomide does not appear to increase the risk of progression in lower risk myelodysplastic syndromes with 5q deletion. A comparative analysis by the Groupe Francophone des Myelodysplasies. *Haematologica*.

[29] Fenaux P, Giagounidis A, Selleslag D (2011). A randomized phase 3 study of lenalidomide versus placebo in RBC transfusion-dependent patients with Low-/Intermediate-1-risk myelodysplastic syndromes with del5q. *Blood*.

[30] Davies F, Baz R (2010). Lenalidomide mode of action: linking bench and clinical findings. *Blood Reviews*.

[31] Quach H, Ritchie D, Stewart AK (2010). Mechanism of action of immunomodulatory drugs (IMiDS) in multiple myeloma. *Leukemia*.

[32] Palumbo A, Freeman J, Weiss L, Fenaux P (2012). The clinical safety of lenalidomide in multiple myeloma and myelodysplastic syndromes. *Expert Opinion on Drug Safety*.

[33] Mitsiades N, Mitsiades CS, Poulaki V (2002). Apoptotic signaling induced by immunomodulatory thalidomide analogs in human multiple myeloma cells: therapeutic implications. *Blood*.

[34] Chang DH, Liu N, Klimek V (2006). Enhancement of ligand-dependent activation of human natural killer T cells by lenalidomide: therapeutic implications. *Blood*.

[35] Kotla V, Goel S, Nischal S (2009). Mechanism of action of lenalidomide in hematological malignancies. *Journal of Hematology and Oncology*.

[36] Ito T, Ando H, Suzuki T (2010). Identification of a primary target of thalidomide teratogenicity. *Science*.

[37] Zhu YX, Braggio E, Shi C-X (2011). Cereblon expression is required for the antimyeloma activity of lenalidomide and pomalidomide. *Blood*.

[38] Boultwood J, Pellagatti A, McKenzie ANJ, Wainscoat JS (2010). Advances in the 5q-syndrome. *Blood*.

[39] Mitsiades CS (2011). How “immunomodulatory” are IMIDs?. *Blood*.

[40] Salmon SE, Crowley JJ, Balcerzak SP (1998). Interferon versus interferon plus prednisone remission maintenance therapy for multiple myeloma: a Southwest Oncology Group study. *Journal of Clinical Oncology*.

[41] Alexanian R, Weber D, Dimopoulos M, Delasalle K, Smith TL (2000). Randomized trial of *α*-interferon or dexamethasone as maintenance treatment for multiple myeloma. *American Journal of Hematology*.

[42] Shustik C, Belch A, Robinson S (2007). A randomised comparison of melphalan with prednisone or dexamethasone as induction therapy and dexamethasone or observation as maintenance therapy in multiple myeloma: NCIC CTG MY.7. *British Journal of Haematology*.

[43] Berenson JR, Crowley JJ, Grogan TM (2002). Maintenance therapy with alternate-day prednisone improves survival in multiple myeloma patients. *Blood*.

[44] Badros AZ (2010). The role of maintenance therapy in the treatment of multiple myeloma. *Journal of the National Comprehensive Cancer Network*.

[45] Barlogie B, Tricot G, Anaissie E (2006). Thalidomide and hematopoietic-cell transplantation for multiple myeloma. *New England Journal of Medicine*.

[46] Attal M, Harousseau JL, Leyvraz S (2006). Maintenance therapy with thalidomide improves survival in patients with multiple myeloma. *Blood*.

[47] Abdelkefi A, Ladeb S, Torjman L (2008). Single autologous stem-cell transplantation followed by maintenance therapy with thalidomide is superior to double autologous transplantation in multiple myeloma: results of a multicenter randomized clinical trial. *Blood*.

[48] Spencer A, Prince HM, Roberts AW (2009). Consolidation therapy with low-dose thalidomide and prednisolone prolongs the survival of multiple myeloma patients undergoing a single autologous stem-cell transplantation procedure. *Journal of Clinical Oncology*.

[49] Lokhorst HM, Van Der Holt B, Zweegman S (2010). A randomized phase 3 study on the effect of thalidomide combined with adriamycin, dexamethasone, and high-dose melphalan, followed by thalidomide maintenance in patients with multiple myeloma. *Blood*.

[50] Morgan GJ, Jackson GH, Davies FE (2008). Maintenance thalidomide may improve progression free but not overall survival; results from the myeloma IX maintenance randomisation. *ASH Annual Meeting Abstracts*.

[51] Cavo M, Pantani L, Tacchetti P (2009). Thalidomide maintenance in multiple myeloma: certainties and controversies. *Journal of Clinical Oncology*.

[52] Palumbo A, Bringhen S, Rossi D (2010). Bortezomib-melphalan-prednisone-thalidomide followed by maintenance with bortezomib-thalidomide compared with bortezomib-melphalan-prednisone for initial treatment of multiple myeloma: a randomized controlled trial. *Journal of Clinical Oncology*.

[53] Mateos MV, Oriol A, Martínez-López J (2010). Bortezomib, melphalan, and prednisone versus bortezomib, thalidomide, and prednisone as induction therapy followed by maintenance treatment with bortezomib and thalidomide versus bortezomib and prednisone in elderly patients with untreated multiple myeloma: a randomised trial. *The Lancet Oncology*.

[54] Attal M, Lauwers-Cances V, Marit G (2012). Lenalidomide maintenance after stem-cell transplantation for multiple myeloma. *New England Journal of Medicine*.

[55] McCarthy PL, Owzar K, Hofmeister CC (2012). Lenalidomide after stem-cell transplantation for multiple myeloma. *New England Journal of Medicine*.

[56] Palumbo A, Hajek R, Delforge M (2012). Continuous lenalidomide treatment for newly diagnosed multiple myeloma. *New England Journal of Medicine*.

[57] Palumbo AP, Delforge M, Catalano J (2011). Incidence of second primary malignancy (SPM) in melphalan-prednisone-lenalidomide combination followed by lenalidomide maintenance (MPR-R) in newly diagnosed multiple myeloma patients (pts) age 65 or older. *ASCO Meeting Abstracts*.

[58] Dimopoulos MA, Richardson PG, Brandenburg N (2012). A review of second primary malignancy in patients with relapsed or refractory multiple myeloma treated with lenalidomide. *Blood*.

[59] Rossi AC, Mark TM, Jayabalan D (2011). Incidence of second primary malignancies (SPM) after 6-years follow-up of continuous lenalidomide in first-line treatment of multiple myeloma (MM). *ASCO Meeting Abstracts*.

[60] Reece DE, Masih-Khan E, Goswami RS (2010). Incidence and characteristics of secondary myelodysplastic syndrome developing during lenalidomide-based regimens in relapsed and/or refractory multiple myeloma patients. *ASH Annual Meeting Abstracts*.

[61] Mailankody S, Pfeiffer RM, Kristinsson SY (2011). Risk of acute myeloid leukemia and myelodysplastic syndromes after multiple myeloma and its precursor disease (MGUS). *Blood*.

[62] Landgren O, Ma W, Kyle RA, Rajkumar SV, Korde N, Albitar M (2012). Polymorphism of the erythropoietin gene promotor and the development of myelodysplastic syndromes subsequent to multiple myeloma. *Leukemia*.

[63] Dimopoulos MA, Chen C, Spencer A (2009). Long-term follow-up on overall survival from the MM-009 and MM-010 phase III trials of lenalidomide plus dexamethasone in patients with relapsed or refractory multiple myeloma. *Leukemia*.

[64] Zonder JA, Crowley J, Hussein MA (2010). Lenalidomide and high-dose dexamethasone compared with dexamethasone as initial therapy for multiple myeloma: a randomized Southwest Oncology Group trial (S0232). *Blood*.

[65] Landgren O, Thomas A, Mailankody S (2011). Myeloma and second primary cancers. *New England Journal of Medicine*.

[66] Kyle RA, Pierre RV, Bayrd ED (1975). Multiple myeloma and acute leukemia associated with alkylating agents. *Archives of Internal Medicine*.

